# Translating evidence-based treatment for digital health delivery: a protocol for family-based treatment for anorexia nervosa using telemedicine

**DOI:** 10.1186/s40337-020-00328-x

**Published:** 2020-10-09

**Authors:** A. Hambleton, D. Le Grange, J. Miskovic-Wheatley, S. Touyz, M. Cunich, S. Maguire

**Affiliations:** 1 InsideOut Institute for Eating Disorders, The Boden Collaboration for Obesity, Nutrition, Exercise and Eating Disorders, The University of Sydney, Sydney, Australia; 2 UCSF Weill Institute for Neurosciences, School of Medicine, University of California, San Francisco, San Francisco, California USA; 3 School of Psychology, Faculty of Science, The University of Sydney, Sydney, Australia; 4 The Boden Collaboration for Obesity, Nutrition & Eating Disorders, Faculty of Medicine and Health (Central Clinical School), The University of Sydney, Sydney, Australia; 5 Sydney Health Economics Collaborative, Sydney Local Health District, Camperdown, NSW Australia; 6 Sydney Local Health District, NSW Health, St Leonards, Australia

**Keywords:** Anorexia nervosa, Cost-consequence analysis, Inequalities, Eating disorder, Covid-19, Young people, Telemedicine, Telemedicine, Family-based treatment, Rural health

## Abstract

**Background:**

Family-based treatment (FBT) is an efficacious outpatient intervention for young people diagnosed with Anorexia Nervosa (AN). To date, treatment to protocol has relied on standard face-to-face delivery. Face-to-face therapy is subject to geographic, temporal and human factors, rendering it particularly susceptible to inequities and disruption. This has resulted in poorer service provision for rural and regional families, and recently a significant challenge to providing face-to-face services during the COVID-19 global pandemic. The present study examines whether FBT for AN can be successfully translated to a digital delivery platform to address these access issues.

**Method:**

Forty young people aged 12 to 18 years who meet DSM-5 diagnostic criteria for AN, and live in a rural or regional setting, will along with their family be recruited to the study. Trained therapists will provide 18 sessions of FBT over 9 months via telemedicine to the home of the young person and their family. The analysis will examine treatment effectiveness, feasibility, acceptability, and cost-effectiveness.

**Discussion:**

The study addresses the treatment needs of families not able to attend face-to-face clinical services for evidence-based treatment for eating disorders. This might be due to several barriers, including a lack of local services or long travel distances to services. There has been a recent and unprecedented demand for telemedicine to facilitate the continuity of care during COVID-19 despite geographical circumstances. If delivering treatment in this modality is clinically and economically effective and feasible, it will facilitate access to potentially lifesaving, evidence-based treatments for families formerly unable to access such care and provide evidence for the continuity of services when and where face-to-face treatment is not feasible.

## Background

Anorexia nervosa (AN) is a serious psychiatric disorder, with numerous negative physical and psychological health impacts [1–3]. It is the third most common chronic disorder affecting adolescent girls [4], although AN affects both genders and is present across the entire lifespan [5]. Children and adolescents are especially vulnerable to the physical consequences of AN such as stunted growth, poor bone health, cardiac complications, infertility, and changes to brain structure [6]. The average duration of AN is 5 to 7 years [7], although long-term follow-up evidence suggests that after a 10-year duration of illness, only 30% of individuals are recovered [8]. Individuals diagnosed with AN have approximately 12 times greater risk of death, and a 57 times greater risk of suicide compared to same-age peers [2]. Given the seriousness of the disorder, treatment must be delivered efficiently and effectively.

Currently, Family-Based Treatment (FBT) is the most efficacious therapy for adolescents with AN [9–11] recommended by international clinical practice guidelines [12, 13]. FBT has been manualised as a face-to-face outpatient behavioural intervention and emphasises parental support as the primary resource in the recovery of adolescents with AN [14]. Several randomised controlled trials (RCTs) have demonstrated the efficacy of FBT for adolescent AN [10, 15–20]. Remission rates are particularly promising for adolescents with short illness duration [11, 16, 21]. The average remission rate for all participants at the end of FBT is approximately 40% [11, 22, 23]. An average of 75% of young people demonstrate improvement in weight and eating-related symptomatology at end of FBT [16].

There is a concerning inequity in access to eating disorder care for regional and rural populations. The implementation of specialised treatments, such as FBT, is challenged by several factors unique to the rural setting in New South Wales (NSW), Australia, a state with a population spread across a large geographic area. Rural health districts range from 11,335 km^2^ (kms) to 246,676 square kms with a population density that varies between .05 square km per person to .89 square km per person. Regional and rural areas are limited by a lack of support resources (such as support groups, consumer advocacy groups), and a limited number of trained professionals confident in the assessment, diagnosis, and treatment of AN [24, 25], particularly using FBT. Further, stigma and concomitant treatment avoidance are amplified by social visibility in small rural communities [26].

There are additional circumstances where available face-to-face treatment sessions are not feasible, such as co-occurring physical or mental health issues, difficulties with travel, families who live at a great distance from each other yet are required to attend sessions (as is the case in some separated families), or natural disasters, such as the recent Australian bushfires which severely impacted regional and rural areas. Furthermore, the recent COVID-19 pandemic forced the sudden shutdown of face-to-face services due to health risks and an anticipated increase in demand for inpatient services [27, 28]. Thus, rapid transition and adoption of new service delivery modalities are urgently required. There is an imperative need to demonstrate how evidence-based therapies, such as FBT, can be successfully delivered over digital platforms [29–31].

Technology, and specifically the use of telemedicine (or telehealth), appears to be an obvious solution [32]. The uptake of telemedicine in the treatment of eating disorders in regional and rural Australia before COVID-19 has been relatively slow [33], given the amount of research on the use of this mode of intervention for psychosocial treatment in other mental health diagnoses [34], such as adolescent ADHD [35] and post-traumatic stress disorder [36]. A systematic review demonstrated some positive findings for e-therapies in eating disorder treatment [37], and results from an RCT of Cognitive Behaviour Therapy-Enhanced (CBT-E) for Bulimia Nervosa (BN) delivered via telemedicine compared to face-to-face treatment demonstrate the need for further exploration of digital treatment delivery [38]. Further, a recent pilot study conducted in the USA found that FBT delivered via telemedicine is feasible, acceptable, and had equivalent outcomes to treatment delivered in traditional face-to-face therapy [14, 39, 40]. However, this pilot research involved a small sample size, treatment delivered was within a specialist eating disorder service, and with only one highly trained therapist. Consequently, little is known about the scalability and ‘real-world’ application of using telemedicine in the delivery of FBT. That is, within an already existing, non-specialist service, directly into the family home. Moreover, there is no evidence to date demonstrating the cost-effectiveness of FBT delivered using telemedicine compared with standard care (i.e. face-to-face care).

### Specific aims

The specific aims of the present study are to determine:

1. The effectiveness of FBT delivered via telemedicine in reducing core eating disorder symptomology, as well as general mood and anxiety, and improving quality of life to young persons with AN and their families living in regional and rural NSW, Australia;
2. The feasibility and acceptability of using telemedicine into the home to deliver FBT to young persons with AN and their families; and
3. The economic costs and health consequences (clinical and quality of life outcomes) of using telemedicine to deliver FBT directly into the home.

## Method

### Overall study design

**Fig. 1:**
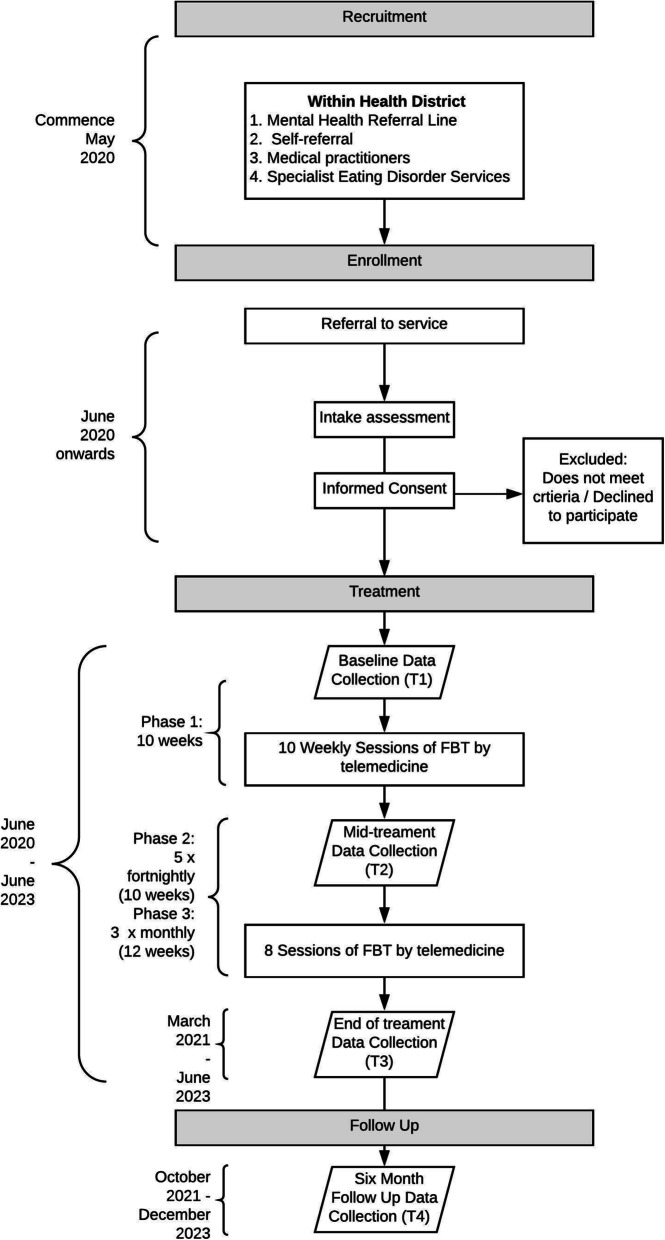
Study timeline for Family-based treatment for Anorexia Nervosa using Telemedicine (May 2020 – December 2023). Note: FBT = family-based treatment

This is a four-year pre- and post-implementation study, with 2 years dedicated to treatment delivery, and up to 20 families per year, to reach a total sample size of 40 families. The study commenced in April 2020 (see Fig. 1 for the Study Timeline). The study protocol has been approved by a Human Research Ethics Review Board (HREC #2020/ETH00186).

### Setting

A secure online telemedicine platform will be used to connect the therapist located at the regional or rural mental health service to the family, who will be in their home.

### Participants

Young people, aged between 12 and 18 years who meet diagnostic criteria for AN as defined by the fifth edition of the *Diagnostic and Statistical Manual of Mental Disorders (DSM-5)* [41] will be recruited for this study. Given the importance of involving the whole family in treatment, the parents/guardians responsible for the young person’s recovery as well as siblings and any other family members involved in treatment will also be recruited. Participants will be a resident of a regional or rural health district in NSW, Australia. The reason for selecting regional and rural health districts is due to the already great need for specialist care in these regions given eating disorder services are sparse in usual service delivery circumstances. Primarily, participants will be recruited via the already existing referral pathways for mental health service for young people, such as via medical practitioners, schools, and local mental health services.

Three therapists from each of the five regional and rural health districts eligible for the study have been recruited (*n* = 15). Each therapist has the capacity to work with *n* = 2 families each year, with a maximum of *n* = 4 families during the study. The therapists providing treatment will contribute to the evaluation, and thus will be enrolled as participants. All therapists had to meet a minimum level of training and experience in FBT, obtain management support to enrol in the study, and commit to participate in additional training and regular supervision.

### Inclusion/exclusion criteria

The young person with AN must be medically stable for outpatient treatment verified by their medical professional [42]. The family must be committed to engaging in FBT, not be engaged in any other psychological or dietetic treatment for AN, have access to a computer or tablet device with videoconferencing functions, a reliable internet connection, access to a telephone or mobile phone if internet unavailable, and access to a reliable digital weighing scale at home. The study can provide some of these requirements to families to ensure equity of inclusion. Young people who require inpatient treatment for a physical or psychiatric illness, are dependent on alcohol or other substances, have acute suicidality, currently pregnant, or under compulsory treatment orders, are excluded from participating in this study.

### Videoconferencing platform

Picture and sound quality are of critical importance, and the investigators want to ensure technical issues do not compromise the therapeutic experience of the patient, family, and therapist. The videoconferencing platform facilitating treatment delivery has end-to-end encryption, integration with digital calendar platforms, password protection and can be used on any device. Video quality will depend on the speed of the family’s internet and the quality of their computer camera. In case the secure connection is lost or cannot be made, the therapist will attempt to reconnect via videoconferencing or call the family via phone.

### Therapist training

Therapists enrolled in the study will receive formal face-to-face training in FBT and using telemedicine from one of the investigators (DLG). DLG was senior author on the previously described USA pilot study [14, 39], has strong links to the training of FBT within Australia [10, 43], and co-authored the FBT treatment manual, *Treatment Manual for Anorexia Nervosa: A Family-based Approach* [44]. Training topics include advanced FBT skills, running the family meal session via videoconferencing, session structure and phases of therapy.

When the treatment delivery phase commences, the therapists will receive weekly group supervision with DLG. The supervision group will take place online using a videoconferencing platform. Focus areas of the consultation group will be largely dependent on the treatment progress of the therapists’ cases; however, likely topics will include treatment adherence, advanced FBT skills, moving through the phases of FBT, and managing therapeutic processes via telemedicine.

### Intervention

Eighteen sessions of FBT will be delivered over 9 months, as described in Lock and Le Grange [44], via telemedicine, to young people with a diagnosis of AN and their families. The key difference to standard FBT is the delivery modality, being video conferencing, and treatment setting, being directly into the family home, rather than face-to-face within the service. The young person’s medical and psychiatric health will be managed by their local general practitioner and/or psychiatrist.

After consent and before meeting with the therapist, a member of the research team will contact the family to perform a technology check, to troubleshoot any technology issues (i.e., video camera not working, teleconferencing platform issues or connection/speed issues), and offer education and training support concerning the videoconferencing platform. Before commencing therapy, the family will have a brief online session with the therapist. The purpose of this session is to orientate the family to the treatment format, describe the treatment process and discuss logistics that will enhance the therapy experience.

Before each scheduled session, the young person will be weighed using the same scales and in the same location of the house by the parent/guardian. As soon as the parents/guardians have measured the young person’s weight, they will send the weight to the therapist via email or text. As per the FBT manual [44], the young person will be weighed wearing light indoor clothing, no shoes and after voiding their bladder. The parents/guardians will be instructed by the therapist how to do this during the first session. The therapist will initiate the therapy session, and begin with an individual meeting with the young person [39, 44]. Following a review of the weekly weight and check in with the young person, the remaining family members will join the young person and therapist.

FBT comprises of three treatment phases [44, 45]. In phase 1, the parents are charged with the responsibility of managing eating behaviours and weight gain. In phase 2, parents gradually transition the responsibility of eating back to the adolescent in an age-appropriate manner. In phase 3, adolescent developmental issues (such as peer friendships or managing school/studies) become the focus of treatment. Due to the seriousness of AN, FBT requires all family members to attend sessions [44], and that appointments are at least weekly for the first several months [45]. This frequency of appointments generally reduces as the young person becomes progressively well [44].

### Fidelity

Treatment fidelity will be managed by therapists completing two workshops and weekly supervision with DLG. Furthermore, therapists will complete a brief checklist after each session and will use these to guide supervision discussions.

### Measures

Psychometric questionnaires will be utilised throughout treatment for research purposes and to monitor treatment progress. There will be several assessment points: baseline (T1), throughout treatment, mid-treatment (T2), end-of-treatment (T3), and at 6 months follow-up (T4) (see Table 1 for assessment schedule). In addition to psychometric questionnaires, the young person, parents/guardians and therapist will be invited to participate in a semi-structured interview at T3. The young person, parents/guardians, siblings/other family members and therapist will complete the assessments via a secure online data capture portal, REDCap. REDCap is a secure web application for building and managing online surveys and databases which is licenced to the University of Sydney. REDCap is compliant with just about any privacy and security standard – for example, HIPAA, Part-11, and FISMA standards (low, moderate, or high).

**Table 1:** Assessment Battery and Schedule

	Baseline (T1)	Weekly (until T3)	Mid-Treatment (Session 9 (T2)	End of treatment (Session 18) (T3)	6-month Follow-up (T4)
*Demographics: age, gender, education, location etc*	X				
*Illness characteristics: duration, symptoms*	X				
*Wait time to commence FBT*	X				
*Resource use in HE: Hospital admissions (number and duration)*	X		X	X	X
**Young Person**					
*Weight (% mBMI)*	X	X	X	X	X
*Eating Disorder Examination (EDE)*	X		X	X	X
*Eating Disorder Examination – Questionnaire (EDE-Q)*	X		X	X	X
*Clinical Impact Assessment (CIA)*	X		X	X	X
*Compulsive Exercise Test (CET)*	X			X	X
*Family Assessment Device (FAD)*	X		X	X	X
*Revised Children’s Anxiety and Depression Scale (RCADS)*	X		X	X	X
*Rosenberg Self Esteem Scale*	X		X	X	X
*EQ-5D-5L* or *EQ-5D-Y*	X		X	X	X
*Ongoing treatment post T3*					X
*Evaluation of treatment*				X	
**Parents/Guardians**					
*Family Assessment Device (FAD)*	X		X	X	X
*Parents* Versus *Anorexia Scale (PVA)*	X	2, 4, 6	X	X	
*Parent Eating Disorder Examination (PEDE)*	X		X	X	X
*Parent Eating Disorder Examination – Questionnaire (PEDE-Q)*	X		X	X	X
*Revised Children’s Anxiety and Depression Scale (RCADS) - Parent*	X		X	X	X
*EQ-5D-5L*	X		X	X	X
*Evaluation of treatment*				X	
**Siblings/Other Family Members**					
*Family Assessment Device (FAD)*	X		X	X	X
*Evaluation of treatment*				X	
*EQ-5D-5L* or *EQ-5D-Y*	X		X	X	X
**Therapist**					
*Session attendance*		X			
*Evaluation of treatment acceptability and feasibility*				X	
**Additional Health Economic Assessments**					
Healthcare resources used by young people with AN (completing a purpose-built cost questionnaire)	X		X	X	X
Non-healthcare costs incurred by young people with AN and their parents/siblings	X		X	X	X
Resources used for implementation of the FBT telemedicine intervention				X	

*%mBMI* percent median Body Mass Index, *HE* health economics

### Demographics, treatment and illness history

Demographic information will be collected to describe the young person (e.g., age, gender) and the family (e.g., socioeconomic status, employment status). The history of treatment (e.g., previous treatment and hospital admissions) and illness (e.g., duration of illness) will also be collected.

### Young person

*Percent Median Body Mass Index (%mBMI):* Weight and height will be collected on the family’s calibrated digital scales. Weight will be measured in kilograms before every treatment session, while height will be collected at T1, T2, T3 and T4. As this study is taking place via telemedicine, the weight will be taken in the young person’s home by the parents/guardians [14]. Percent median body mass index (%mBMI) will be calculated using the Centres for Disease Control (CDC) [46] charts for height, weight, gender, and age. Full symptom remission in this study greater than 85%mBMI, and an Eating Disorder Examination (EDE) score [47] within two standard deviations of population means [22].

*Eating Disorder Examination* (EDE [48];) is a standardised interview that measures the severity of eating disorder symptomology. It is a measure of present state, and, assesses both the frequency of key behaviours (including various forms of overeating and purging) and the severity of psychopathology along certain dimensions (dietary restraint, concern about eating, concern about shape, and concern about weight).

*Eating Disorder Examination – Self-Report Questionnaire* (EDE-Q [49];) is a self-report measure that was adapted from the EDE [47]. The EDE-Q is a measure of present state and, except for the items that pertain to diagnosis, it is exclusively concerned with the preceding 4 weeks. The EDE-Q assesses both the frequency of key behaviours and the severity of psychopathology along certain dimensions (dietary restraint, concern about eating, concern about shape, and concern about weight).

The *Clinical Impairment Assessment Questionnaire* (CIA [50];) is a 16-item self-report measure that measured the young person’s psychosocial impairment associated with eating disorder symptoms. The results of the CIA are particularly useful when used in conjunction with an eating disorder specific measure covering the same 28-day timeframe, like the EDE-Q.

The *Compulsive Exercise Test* (CET [51];) assesses five core features of compulsive exercise: avoidance and rule-driven behaviour, weight control exercise, mood improvement, lack of exercise enjoyment and exercise rigidity. The CET is included as excessive exercise is a common feature of an eating disorder.

The *Family Assessment Device* (FAD [52];) is a 60-item self-report measure that explores family functioning in seven subscales: problem-solving, communication, roles, affective responsiveness, affective involvement, behaviour control, and general functioning. There is evidence of a relationship between family functioning, communication, and problem-solving subscales and remission at the end of FBT [53].

The *Revised Children’s Anxiety and Depression Scale* (RCADS [54];) is a 47-item self-report questionnaire with six subscales: separation anxiety disorder, social phobia, generalised anxiety disorder, panic disorder, obsessive-compulsive disorder, and major depressive disorder plus a total internalising scale. The RCADS has been used in studies of young people with eating disorders [15] as a screener for comorbidities.

The *Children’s Global Assessment Scale* (CGAS [55, 56]) is a rating of functioning for children and young people aged 6–17 years old. The young person is given a single score between 1 and 100, based on a range of aspects related to psychological and social functioning. The score ranges from ‘extremely impaired’ to ‘doing very well’.

The *Rosenberg Self-Esteem Scale* (RSES [57];) is a 10-item self-report measure of an individual’s overall self-esteem. Low self-esteem is often observed in young persons with AN [58].

The *EQ-5D-Y* [59] or the *EQ-5D-5L* [60] (depending on the young person’s age) will be used to evaluate health and quality of life. The *EQ-5D-Y* is validated for young people up to the age of 15 years. For those older than 15 years, the EQ-5D-5L will be used.

The young person’s *Evaluation of treatment* will be completed via an online survey and semi-structured interview [14]. The semi-structured interview will explore the young person’s experience of FBT [61], barriers and challenges when receiving treatment via telemedicine and suggestions and considerations for future implementation.

### Parent/Guardian

Given the crucial role of parents/guardians in the success of FBT, a parent/guardian rating of the young person’s eating disorder symptomology and mental health status will be obtained.

*Parent Version Eating Disorder Examination* (PEDE [62],) is an interview-based version of the EDE for parents/carers [48]. The PEDE asks for parental perspective in the recent history of their child’s eating concerns, with questions formatted similarly to the EDE. It assesses both the frequency of key behaviours (including various forms of overeating and purging) and the severity of psychopathology along certain dimensions (dietary restraint, concern about eating, concern about shape, and concern about weight).

The *Parent Version of the Eating Disorder Examination Questionnaire* (PEDE-Q) [62], is a self-report questionnaire that will be used to obtain the parents’ rating of their child’s symptoms. The PEDE-Q has four subscales: restraint, eating concern, shape concern and weight concern.

The *Family Assessment Device* (FAD [52];) is a 60 item self-report measure that explores family functioning in seven subscales: problem-solving, communication, roles, affective responsiveness, affective involvement, behaviour control, and general functioning.

The *Parent* Versus *Anorexia Scale* (PVA) [63] is a seven item measure of self-efficacy of parents/guardians participating in FBT. It assesses the parent/guardians’ belief in their ability to facilitate recovery in their child and is commonly used in eating disorder research [15, 64].

The *Revised Children’s Anxiety and Depression Scale – Parent Version* (RCADS-P) [54] is a modified version of the RCADS, also with 47-items and six subscales.

The *EQ-5D-5L* [60] will be used to assess the general health and quality of life of the parent/guardians.

*Parent/Guardian evaluation of treatment* will be assessed using a short online survey and semi-structured interview to gather information from the parents regarding their opinions of the intervention and mode of delivery and will be used to determine what aspects of the treatment and telemedicine were most helpful and least helpful [14]. The semi-structured interview will explore the parent/guardian’s experience of using telemedicine to receive FBT, what they liked and did not like, barriers and challenges, perceived advantages, and suggestions and considerations for future implementation. Further, the interview will explore what factors (themes) contribute to the effectiveness of FBT and the richness of the family therapy experience, both of which can be difficult to capture in purely quantitative measures.

### Siblings/other family members

The *Family Assessment Device* (FAD [52];) is a 60-item self-report measure that explores family functioning in seven subscales: problem-solving, communication, roles, affective responsiveness, affective involvement, behaviour control, and general functioning.

*Sibling/Other family members evaluation of treatment* will be assessed using a short online survey to gather information regarding their experience of telemedicine were most helpful and least helpful as designed by the researchers for this study [14].

Depending on the sibling/other family member’s age, the *EQ-5D-5L* [60] (over 15 years) or *EQ-5D-Y* [59] (under 15 years) will be used to measure the general health and quality of life.

### Therapist

The therapist will note *Session attendance* of family members every session.

The *Therapist evaluation of feasibility and acceptability* will be measured using a brief online questionnaire and a semi-structured interview after treatment delivery for each family to explore the therapist’s experience of being in the study and delivering FBT via a telemedicine modality. In particular, the semi-structured interview will explore the therapist’s experience of utilising telemedicine to deliver FBT, what components of the alternate mode of delivery enhanced their clinical practice, what were the barriers and challenges, and suggestions and considerations for future implementation.

### Health economics

The participants’ health-related quality of life will be collected using the EQ-5D measures noted above. Resource use collected through the costs and consequences analysis will focus on those healthcare resources associated with the FBT telemedicine intervention or standard care (face-to-face), including the cost of training staff, time costs of health professionals seen by participants for this treatment (therapists and coordinators), laboratory costs, hospitalisations, GP visits, specialist visits, allied health other than through the study, and prescribed treatments and medications (i.e. a micro-costing approach). This resource use data will be collected using purpose-built cost questionnaires administered at T1, T2, T3 and T4. Unit costs will be obtained from a combination of market process (e.g. hourly wage rate), published data and estimates provided by individuals (especially for out-of-pocket costs).

### Primary outcomes

To determine treatment effectiveness (Aim 1), an increase in %mBMI to greater than 85% will be used as an indicator of remission from pre-treatment (T1) to post-treatment (T3) and pre-treatment (T1) to six-month follow-up (T4). In addition, changes from T1 to T3 and T1 to T4 in eating disorder symptomology (global score on the EDE/EDE-Q and PEDE/PEDE-Q), exercise behaviours (CET) and impairment (CIA); general mental health (RCADS and RCADS-P); quality of life (EQ-5D-5L and EQ-5D-5L-Y); family functioning (FAD); and, parental self-efficacy (PVA).

### Secondary outcomes

Treatment acceptability and feasibility (Aim 2) will be determined by the quantitative and qualitative data obtained from online surveys and semi-structured interviews with the young person, family members and therapist. A range of health economic assessments will also be conducted, including quantifying the resources used (costs) and consequences associated with FBT telemedicine or standard care (face-to-face) from a societal perspective; longitudinally examining the impact of socioeconomic, demographic and illness factors on health-related quality of life; and assessing inequalities in access to healthcare for young people with AN during the study (Aim 3).

### Data analysis

#### Aim 1: Effectiveness of delivering FBT via telemedicine

A mixed model logistic regression will be used to evaluate the effectiveness of FBT delivered via telemedicine, using change of %mBMI as the primary outcome from T1 to T3 and from T1 to T4. To determine the effectiveness, secondary analyses, again using a mixed model regression, will compare the young persons’ global EDE and EDE-Q, as well as the parents/guardians PEDE and PEDE-Q scores. Further, the young persons’ responses on the CIA, CET at T1, T2, T3 and T4 will be compared. The young persons’, parent/guardians, and sibling/other family members responses on the FAD and EQ-5D-5L/EQ-5D-Y in addition to the parent/guardian responses on the PVA will be analysed to determine changes in family functioning, parental efficacy, and wellbeing from T1 to T3 and T1 to T4.

#### Aim 2: To determine the feasibility and acceptability of using telemedicine to deliver FBT

To examine the feasibility and acceptability of using telemedicine to deliver FBT we will utilise a mixed-method approach to analyse: (a) the number of patients/families that expressed interest in participating in the study; (b) the number of patients/families enrolled in the study (i.e., an indicator of treatment feasibility); (c) the number and percent of patients/families that completed at least 10 sessions (i.e., an indicator of treatment engagement); (d) the number of patients/families completing the full course of treatment (18 sessions; i.e., an indicator of treatment retention); (e) the results from the post-treatment evaluation survey and semi-structured interview by family members (i.e., family acceptability); (f) the results from the post-treatment evaluation survey and semi-structured interview by young people (i.e., young person acceptability); and (g) the results from the post-treatment evaluation survey and semi-structured interview by therapists (i.e., therapist acceptability).

A qualitative analysis will examine the participants’ responses to the semi-structured interviews that take place at T3. Given the lack of theory or framework regarding family acceptability and feasibility using telemedicine, a conventional content analysis method will be used [65]. The interviews will be analysed within and between participant roles (i.e., young persons, parents/guardians, siblings/other members and therapists) to determine what themes emerge that are similar across the groups, as well as those unique to each group. Two investigators (AH and JMW) with experience in qualitative research will immerse themselves in the data and allow for categories and names for categories to emerge from the data.

#### Aim 3: Health economic assessments

The health economic assessments will consist of three analyses. Firstly, a costs (resources used) and consequences (health outcomes) analysis in which all the direct (medical) costs, indirect costs (e.g. proportion of total internet usage, parents’ time off work to engage in the intervention) and a catalogue of health outcomes (including clinical and health-related quality of life measures) between T1 and T3, and T1 and T4 will be quantified and reported. A societal perspective will be adopted for this analysis, enabling consideration of costs and consequences related to the young person with AN, their family members/carers and the therapist. A resource use database will be developed to collect information on type, amount and value (in dollars) of the healthcare resources associated with the FBT intervention via telemedicine or standard care (face-to-face mode), including the cost of staff training, time costs of the therapist to deliver FBT, laboratory costs, hospitalisations, GP visits, specialist visits, allied health other than through the study, and prescribed treatments and medications. In addition, out-of-pocket costs such as time cost of internet in the home and time off work for parents to engage in the intervention will be collected. This resource use data will be collected using detailed cost questionnaires administered at baseline (T1), T2, T3 and follow-up (T4).

The second analysis will examine the impact of socioeconomic, demographic and illness factors on changes in quality of life (e.g. using the overall score and scores for the unique domains in the health-related quality of life measures for patients, EQ-5D) in the young person and their family members/carers over time using longitudinal data methods. In particular, a multi-level random coefficient model will be used to analyse repeated measures of the health-related quality of life of young people with AN at the four questionnaire time points [66, 67]. Questionnaire point (time) will be included in all models to account for changes in health-related quality of life over time. The models will also be adjusted for the effects of socioeconomic status (e.g. school leaving age, highest qualifications and current or last occupation of parents; young person’s educational attainment to date; and income bracket of the family), demographics (e.g. age, identified gender and area of residence) and illness factors (e.g. years since first diagnosed with AN, severity, co-occurring health conditions).

The third analysis will involve assessing changes in inequalities in access to care sought through the collection of demographic data as well as data on health care need, based on the severity and duration of illness, how and what services have been sought, reach, the use of health care services and the degree to which need for services has been met by this telemedicine intervention [68].

## Discussion

This will be the first Australian study to determine the effectiveness, feasibility, acceptability, and establish preliminary estimates of the health economics of FBT delivered via telemedicine. The COVID-19 pandemic triggered a rapid transition to telemedicine and highlighted the crucial need for assessment of this modality. Should the results demonstrate that FBT can be delivered effectively via telemedicine, the implications for those unable to attend face-to-face sessions whatever the reason are considerable, particularly for those in regional and rural locations where there are obstacles of distance and a lack of local specialist services. Further, given the seriousness of AN, and long-term health consequences, there is an imperative to ensure that evidence-based treatment is delivered to young people as efficiently and effectively as possible.

There is a strong evidence base for FBT as an effective treatment for AN in young people. A large body of this research has taken place in specialist face-to-face services, located in populated, urban locations [10, 15, 18, 20, 21]. The results from the USA pilot study [14] using telemedicine to deliver FBT to rural families were promising, showing FBT delivered via telemedicine was as effective as face-to-face treatment. That being said, this study was based on a small sample, with one expert FBT clinician, and within a specialist eating disorder service. The real-world translation of this finding is yet to be seen; that is, across a large scale, with non-expert (but trained and supported) clinicians and within non-specialist services.

With regards to measuring treatment fidelity, the sessions will not be recorded and reviewed retrospectively by an independent expert. It was deemed more appropriate to manage fidelity in an agile way, via therapists being provided with and using the detailed treatment manual [44], attending two advanced workshops and ongoing regular supervision with investigator and co-author of the manual DLG, and completing a self-report checklist each session rating their compliance with targeted session goals. This checklist can be utilised within supervision and therefore, corrections and adjustments to treatment fidelity can be made immediately, therefore ensuring the family and young person are receiving the correct treatment dosage and method.

This study will provide quantitative data regarding the effectiveness of FBT via telemedicine on eating disorder and other relevant outcomes. However, the study will also provide important qualitative insights into the experiences of delivering and receiving treatment for AN via telemedicine during an unprecedented time. The interviews will explore the technical, ethical, and practical issues of delivering therapy via telemedicine, both in general and also when working with more than one client (i.e., with families). Further, the interviews will be able to capture the complexity of the family’s experience receiving FBT [43, 61, 69]. Further, the health economic assessments will involve (1) identifying, measuring and valuing both the costs and the consequences of FBT telemedicine or standard care (face-to-face); (2) assessing how changes in health-related quality of life are associated with demographic, socioeconomic and illness-related measures; and (3) an assessment of changes in inequalities in access to healthcare over time for young people with AN. The health services change required to meet the needs of regional and rural populations must not only be effective in reducing core eating disorder symptomology but also acceptable and cost-effective.

To begin with, we are focusing recruitment on regional and rural locations, as there has always been an increased need for telemedicine in these areas [24, 25], which is anticipated to increase over the next 2 years due to COVID-19 [14, 30, 31, 34, 39, 40, 70]. Should the study demonstrate that telemedicine is effective, acceptable and feasible for this geographical group, then clinicians can consider the modality as a viable method to effectively disseminate treatment to families not able to attend face-to-face sessions for a multitude of reasons. The COVID-19 pandemic forced therapists and families to rapidly adjust to online modes of treatment delivery. This may help telemedicine establish a more permanent place within health service frameworks and service delivery models. However, further understanding of the differences between online and face-to-face treatment is required to ensure that telemedicine is safe, effective and that young people diagnosed with AN can access evidence-based care, irrespective of their location.

## Data Availability

Not applicable.

## Abbreviations

AN: Anorexia Nervosa
FBT: Family Based Therapy
LHD: Local Health District
EDC: Eating Disorder Coordinator
RCT: Randomised Clinical Trial
NSW: New South Wales
HREC: Human Research Ethics Committee
DSM-5: Diagnostic and Statistical Manual of Mental Disorders, fifth edition
GP: General Practitioner
TAU: Treatment as Usual
BMI: Body Mass Index
%mBMI: Percent Median Body Mass Index
